# Supplementary material for: Crystal structures of aconitase X enzymes from bacteria and archaea provide insights into the molecular evolution of the aconitase superfamily
Source: Commun Biol. 2021 Jun 7;4:687. doi: 10.1038/s42003-021-02147-5 (PMC8184944; doi:10.1038/s42003-021-02147-5)
Supplement: Supplementary file 2 — Supplementary Information [file 42003_2021_2147_MOESM2_ESM.pdf]

Supplementary information

Crystal structures of aconitase X enzymes from bacteria and archaea provide insights into the molecular evolution of the aconitase superfamily

Seiya Watanabe*[‡§¶1], Yohsuke Murase[‡1], Yasunori Watanabe[‡§2], Yasuhiro Sakurai[||], Kunihiko Tajima[||]

[‡] Department of Bioscience, Graduate School of Agriculture, Ehime University, 3-5-7 Tarumi, Matsuyama, Ehime 790-8566, Japan
[§] Faculty of Agriculture, Ehime University, 3-5-7 Tarumi, Matsuyama, Ehime 790-8566, Japan
[¶] Center for Marine Environmental Studies (CMES), Ehime University, 2-5 Bunkyo-cho, Matsuyama, Ehime 790-8577, Japan
[||] Department of Molecular Chemistry, Kyoto Institute of Technology, Matsugasaki, Sakyo-ku, Kyoto, 606-8585

[1] These authors contributed equally to this work.
[2] Present address: Faculty of Science, Yamagata University, 1-4-12 Kojirakawa-machi, Yamagata, Yamagata 990-8560, Japan

* To whom correspondence should be addressed. Tel./Fax: +81-89-946-9848; E-mail: irab@agr.ehime-u.ac.jp

Supplementary methods

Preparation of recombinant ApAcnX and AraC

The primer sequences used are shown in Table S2. The pETDuet-1 vector (Novagen), with two multiple cloning sites (MCS-1 and MCS-2), was used for the cloning and expression of the recombinant ApAcnX protein. The ApAcnX$_L$ (APE_2087.1) and ApAcnX$_S$ (APE_2089) genes were amplified by PCR using primers containing appropriate restriction enzyme sites at the 5'- and 3'-ends and the genome DNA of *Aeropyrum pernix* K1 as a template (kindly gifted from Prof. Hiroyuki Hori (Ehime University)), and were then introduced into MCS-1 (BamHI-HindIII site) and MCS-2 (NdeI-XhoI site), respectively, by which 14 additional residues (MGSSHHHHHHSQDP) were attached at the N terminus of ApAcnX$_L$ (pHisApAcnX). The plasmid of pHisApAcnX was transformed into *E. coli* BL21-CodonPlus(DE3)-RIL cells (Novagen). The overexpression and purification of ApAcnX were performed using the same procedures as TkAcnX. AraC (from *Herbaspirillum huttiense*) was prepared using the same procedures described previously [1].

Reference

1. Watanabe, S., Fukumori, F., Nishiwaki, H., Sakurai, Y, Tajima, K. & Watanabe, Y. Novel non-phosphorylative pathway of pentose metabolism from bacteria. *Sci. Rep.* **9,** 155 (2019).

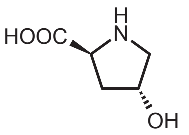

trans-4-Hydroxy-L-proline

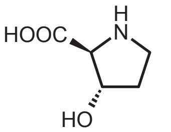

trans-3-Hydroxy-L-proline

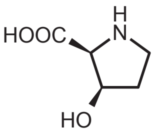

cis-3-Hydroxy-L-proline
(C3LHyp)

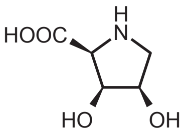

2,3-*cis*-3,4-*cis*-3,4-Dihydroxy-L-proline

Supplementary Figure 1. Chemical structures of L-hydroxyprolines including C3LHyp.

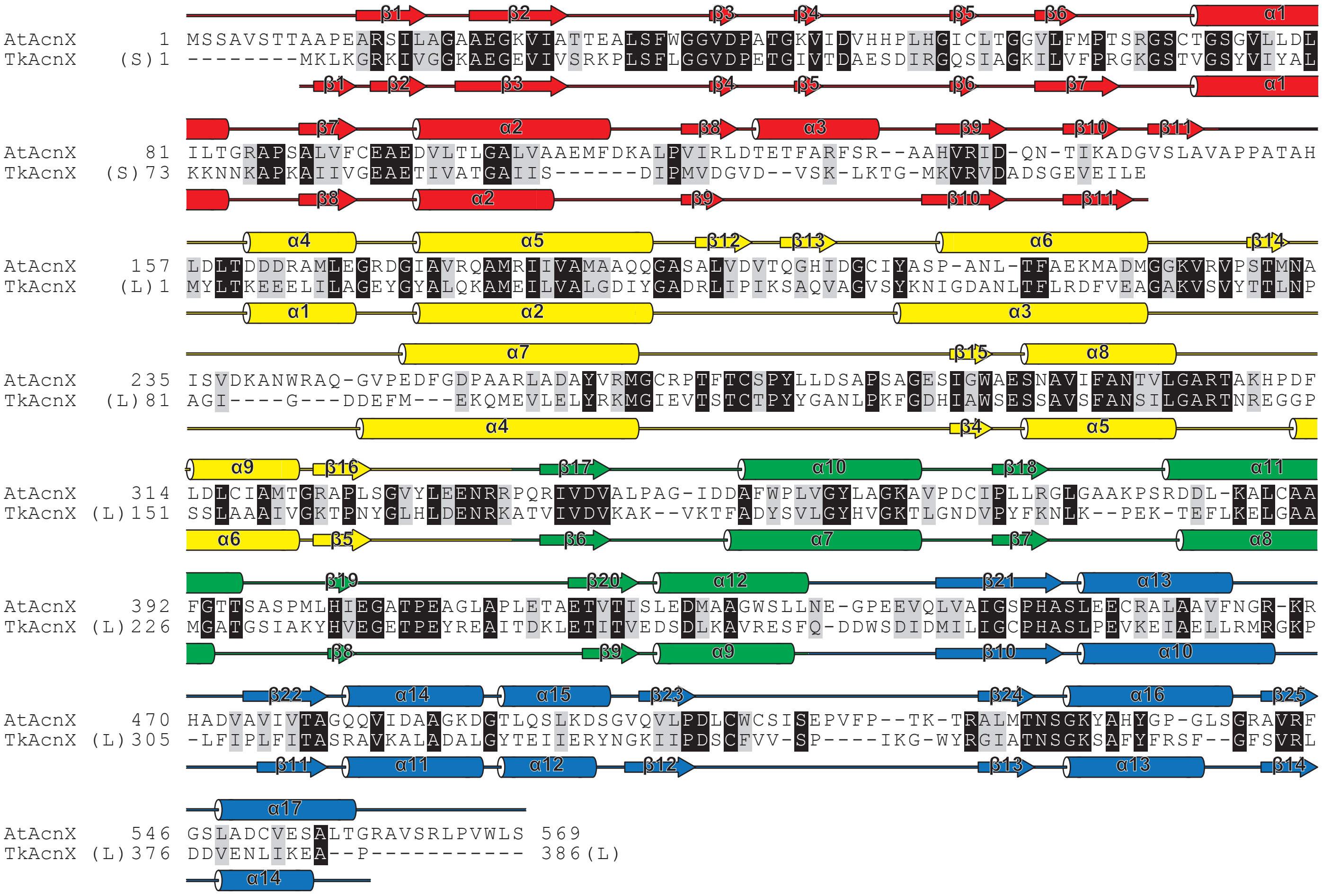

Supplementary Figure 2. Structure-based amino acid sequence alignment of AtAcnX and TkAcnX. Conserved residues are shown as white letters in black boxes. The secondary structural assignments for AtAcnX and TkAcnX, including helices (cylinders) and strands (arrows), are shown above and below the alignment, respectively. The domains are colored as in Fig. 3b, c.

a

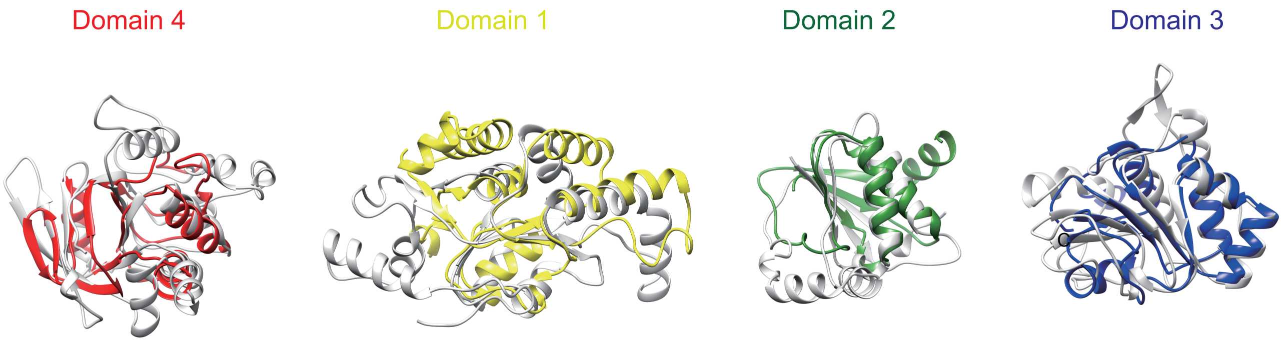

b

mAcn (1C96)	3.1 Å (79/205 Cα)<br>5.1 %	4.0 Å (88/200 Cα)<br>9.1 %	3.0 Å (77/119 Cα)<br>9.1 %	1.9 Å (115/183 Cα)<br>21.7 %
IRP (2B3X)	3.3 Å (76/234 Cα)<br>6.6 %	3.2 Å (111/240 Cα)<br>9.0 %	3.1 Å (74/127 Cα)<br>10.8 %	2.4 Å (113/223 Cα)<br>13.3 %
IPMI (3VBA and 4KP1)	2.6 Å (106/106 Cα)<br>11.3 %	3.7 Å (105/157 Cα)<br>9.5 %	3.1 Å (75/115 Cα)<br>12.0 %	2.4 Å (115/150 Cα)<br>20.0 %
HACN (2PKP and 4KP2)	2.6 Å (108/108 Cα)<br>11.1 %	3.7 Å (100/152 Cα)<br>17.0 %	2.8 Å (74/118 Cα)<br>12.2 %	2.1 Å (99/148 Cα)<br>25.3 %
AcnB (1L5J)	2.9 Å (103/201 Cα)<br>8.7 %	2.7 Å (106/146 Cα)<br>12.3 %	2.8 Å (73/148 Cα)<br>12.3 %	2.2 Å (113/189 Cα)<br>15.0 %

Supplementary Figure 3. (a) Superpositions of AtAcnX domains 1-4 (colored as in Fig. 3b) on mAcn (gray). **(b)** Comparisons of r.m.s.d. (upper values) and structure-based sequence identities (lower values) of domains 1-4 between AtAcnX and other aconitase enzymes (mAcn, IRP, IPMI, HACN, and AcnB).

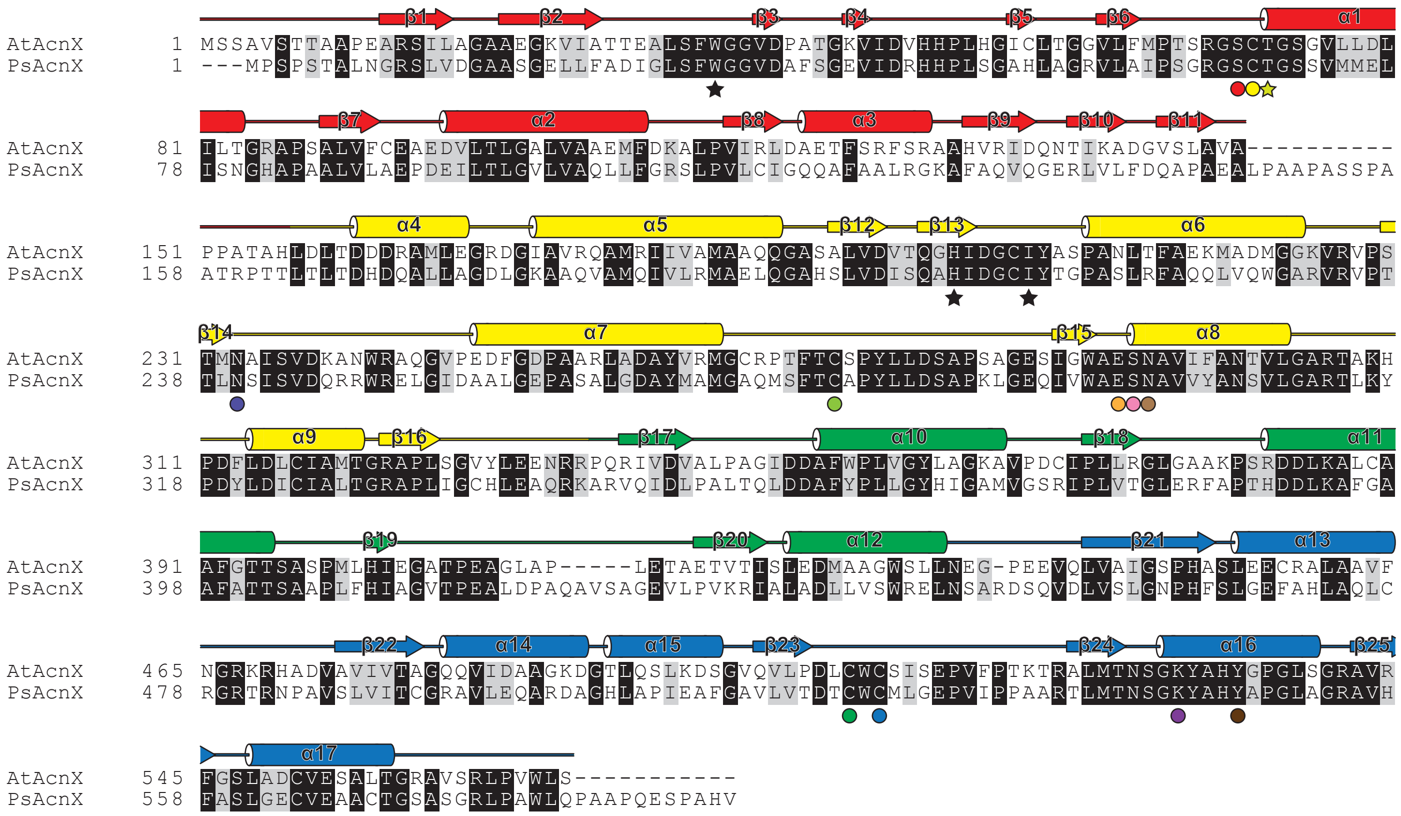

Supplementary Figure 4. Sequence alignment between AtAcnX and PsAcnX. Secondary structures of AtAcnX are shown on the sequence. The domains are colored as in Fig. 3b, Active sites of AtAcnX, shown below the alignment, correspond to Fig. 2b.

	A. tumefaciens (this study)	Relative activity (%)	*P. aeruginosa* (ref. 15)	Relative activity (%)
★	Trp35	1.0	Trp31	-
●	Ser70	0.4	Ser66	0
●	Cys71	-	Cys67	0.4
☆	Thr72	18	Thr68	-
★	Asp203	0.3	Asp205	-
★	Ile206	7.4	Ile208	-
●	Cys273	-	Cys275	0
●	Glu292	-	Glu294	0
●	Ser293	6.7	Ser295	0
●	Cys508	-	Cys516	0
●	Cys510	-	Cys518	0
●	Lys530	0.6	Lys538	0
●	Tyr534	-	Tyr542	0

Supplementary Figure 5. Characterization of alanine mutants of AcnX$_{Type-I}$ of *A. tumefaciens* (this study) and *P. aeruginosa* [15]. Amino acid residues at the 13 sites selected (colored symbols correspond to Fig. 2b) are conserved between them. Relative activity values are expressed as percentages of the values obtained in each wild-type enzyme.

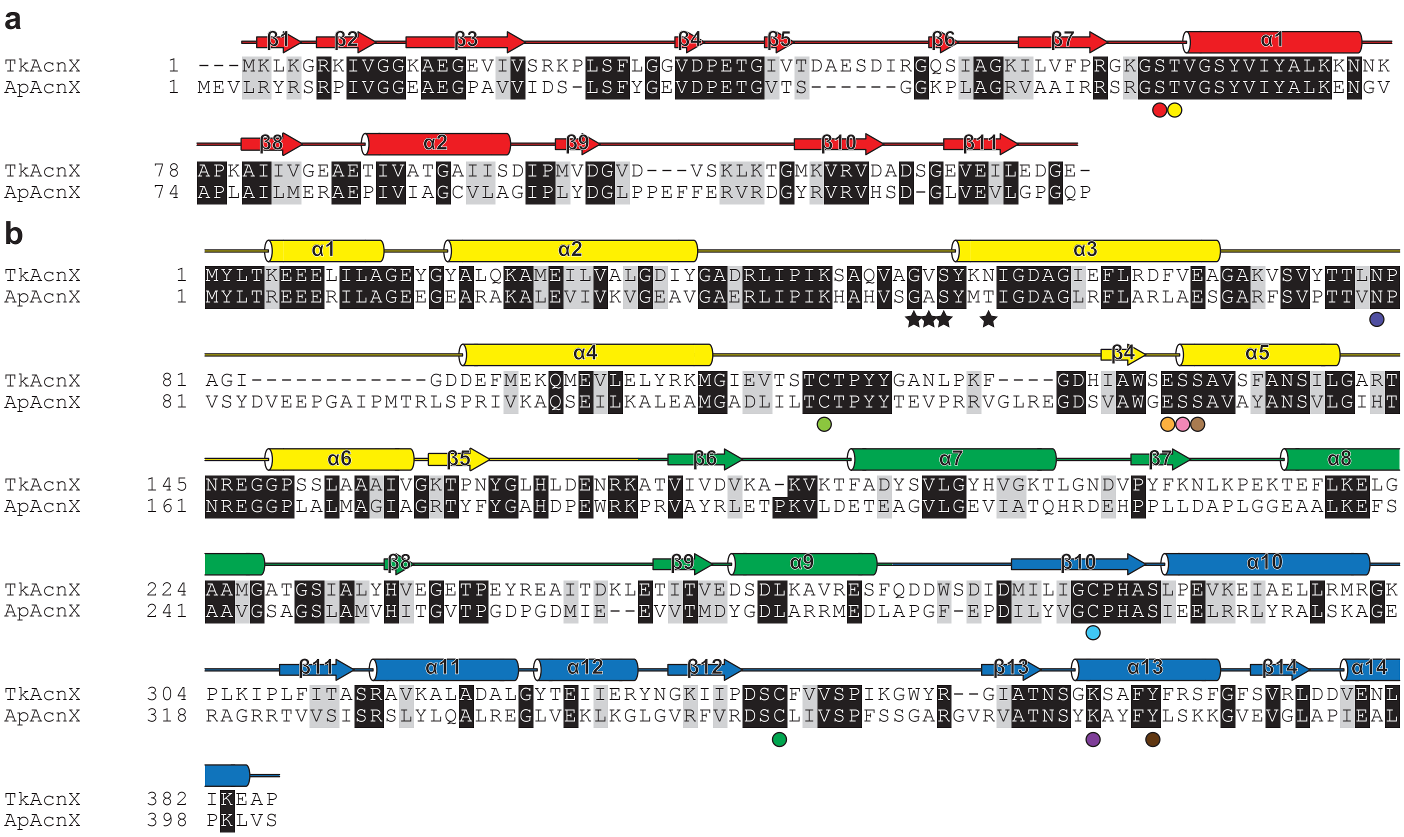

Supplementary Figure 6. Sequence alignment of small (a) and large subunits (b) between TkAcnX and ApAcnX. Secondary structures of TkAcnX are shown on the sequence. The domains are colored as in Fig. 3b, Active sites of TkAcnX, shown below the alignment, correspond to Fig. 2b.

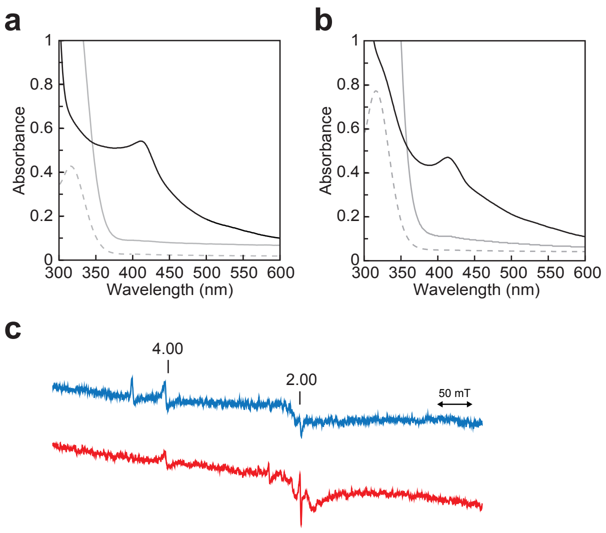

Supplementary Figure 7. UV visible absorption spectra of TkAcnX **(a)** and ApAcnX **(b)** as isolated (black) and following reduction with $Na_2S_2O_4$ (gray). Five-fold diluted sample of the later is shown as dashed line. **(c)** EPR spectra of TkAcnX as isolated (blue) and following reduction with $Na_2S_2O_4$ (red) at 20K.

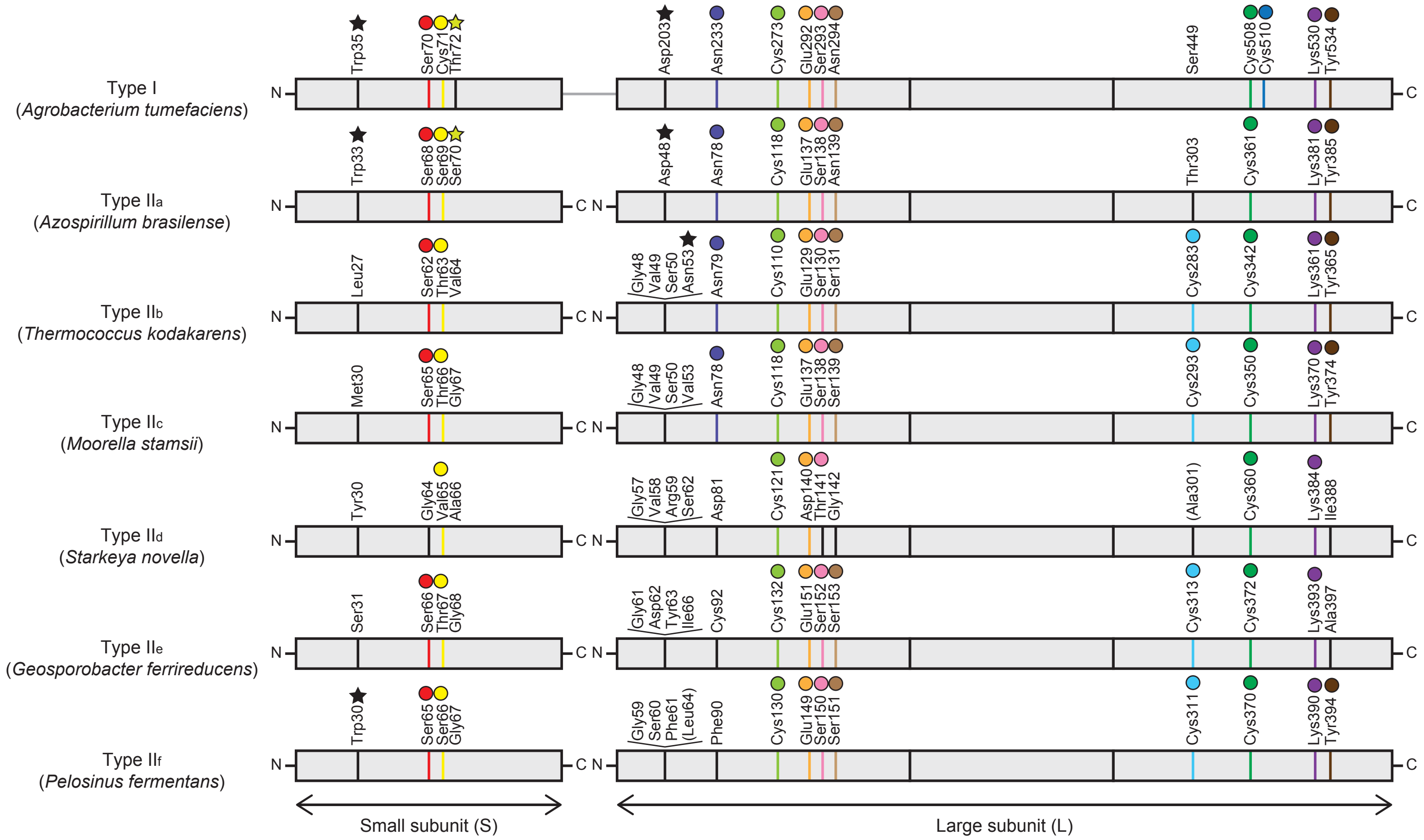

Supplementary Figure 8. Schematic comparisons of (putative) active sites in seven subclasses of AcnX. Amino acid residues with the same symbols are conserved.

Supplementary Table 1. Kinetic parameters for C3LHyp by AtAcnX mutants

Enzyme	K_m (mM)	k_{cat} (min^{-1})	k_{cat}/K_m ($min^{-1}\cdot mM^{-1}$)
WT	1.16±0.11	2000±166	1720±50
W35A	4.00±0.95	2.19±0.06	0.564±0.107
T72A	1.84±0.06	414±13	225±2
I206A	2.37±0.35	134±16	56.8±1.3
S293A	1.27±0.03	85.7±1.5	67.4±0.7

The dehydration activity was assayed spectrophotometrically using a coupling enzyme. Values are the means ± SD, n = 3. Eight different concentrations of the substrate between 0.1 and 1 mM were used.

Supplementary Table 2. Primers used in the present study

Primer	Sequence
Cloning of the *TkAcnX*$_S$ gene into pETDuet-1[1]	
P1 (BamHI)	5′-catggatccgAAGCTCAAGGGCAGGAAGATCGTTGGG-3′
P2 (HindIII)	5′-attaagcttCTACTCCCCATCCTCAAGGATTTCGAC-3′
Cloning of the *TkAcnX*$_L$ gene into pETDuet-1[1]	
P3 (NdeI)	5′-gaccatATGTACCTGACGAAGGAGGAGGAGC-3′
P4 (XhoI)	5′-atgctcgagTCACGGAGCCTCCTTTATCAGGTTCTC-3′
Introduction of the rhinovirus 3C protease cleavage site from humans in TkAcnX	
P5	5′-tttcagggtccgAAGCTCAAGGGCAGGAAGAT-3′
P6	5′-cagcacttccagCGGATCCTGGCTGTGGTGAT-3′
Site-directed mutagenesis in the *AtAcnX* gene[2]	
P7 (W35A-F)	5'-AGCTTCGCCGGCGGTGTCGATCCCGCCACC-3'
P8 (W35A-R)	5'-ACCGCCGGCGAAGCTCAGCGCCTCTGTCGT-3'
P9 (S70A-F)	5'-CGCGGCGCCTGCACCGGCTCGGGTGTGTTG-3'
P10 (S70A-R)	5'-GGCGTATGCGCCGGAATTGGTCATCAGCGC-3'
P11 (T72A-F)	5'-TCCTGCGCCGGCTCGGGTGTGTTGCTCGAT-3'
P12 (T72A-R)	5'-CGAGCCGGCGCAGGAGCCGCGGCTGGTCGG-3'
P13 (D203A-F)	5'-CATATTGCCGGCTGCATCTATGCCAGCCCC-3'
P14 (D203A-R)	5'-GCAGCCGGCAATATGGCCCTGGGTAACATC-3'
P15 (I206A-F)	5'-GGCTGCGCCTATGCCAGCCCCGCCAACCTT-3'
P16 (I206A-R)	5'-GGCATAGGCGCAGCCGTCAATATGGCCCTG-3'
P17 (S293A-F)	5'-GCCGAAGCCAATGCGGTGATCTTCGCCAAT-3'
P18 (S293A-R)	5'-CGCATTGGCTTCGGCCCAGCCGATCGATTC-3'
P19 (K530A-F)	5'-TCCGGCGCATACGCCCATTACGGTCCGGGT-3'
P20 (K530A-R)	5'-GGCGTATGCGCCGGAATTGGTCATCAGCGC-3'
Cloning of the *ApAcnX*$_S$ gene into pETDuet-1[1]	
P21 (NdeI)	cgaccatATGGAGGTCCTCAGGTACAGGTCCAGGCC
P22 (XhoI)	gatgctcgagCTAGGGCTGGCCAGGGCCTAGGACCTCG
Cloning of the *TkAcnX*$_L$ gene into pETDuet-1[1]	
P23 (BamHI)	cccaggatccTTACCTGACGAGGGAGGAGGAGAGGATTC
P24 (PstI)	ttggctgcagCTACGACACCAGCTTTGGCAGAGCCTC
[1] Lower case letters indicate additional bases for introducing the underlined digestion sites of restriction enzymes in parentheses.	
[2] F and R indicate forward and reverse primers, respectively. Underlining indicates mutated regions.	
